# Deliberative dialogue for co-design, co-implementation and co-evaluation of health-promoting interventions: a scoping review protocol

**DOI:** 10.1186/s40900-025-00680-9

**Published:** 2025-02-28

**Authors:** Kian Godhwani, Abimbola K. Saka, Vinesha Ramasamy, Bee-Lee Soh, Mathangee Lingam, Aisha Lofters, Eva Grunfeld, David Gerstle, Peter Selby, Ambreen Sayani

**Affiliations:** 1 Women’s College Research Institute, Women’s College Hospital, Toronto, Ontario Canada; 2 Campbell Family Mental Health Research Institute, Centre for Addictions and Mental Health, Toronto, Ontario Canada; 3 Department of Family and Community Medicine, University of Toronto, Toronto, Ontario Canada; 4 Dalla Lana School of Public Health, University of Toronto, Toronto, Ontario Canada; 5 University of Toronto Mississauga, Library, Mississauga, Ontario Canada

**Keywords:** Deliberative dialogue, Health promotion, Knowledge translation, Patient engagement, Multistakeholder, Interdisciplinary, Health interventions, Co-design, Co-implementation, Co-evaluation

## Abstract

**Introduction:**

Deliberative dialogue (DD) is a participatory research methodology wherein stakeholders with diverse backgrounds, experiences and interests come together to engage in discussions to build consensus for collaborative decision-making. The methodology is increasingly used in health promotion research to develop equitable solutions to complex problems. A review of PubMed-indexed papers alone showed a 9% increase in published DD studies in 2024 from prior years (2020–2023), with most focusing on health promotion and service co-design. Given the increasing emphasis on multistakeholder engagement in research, there is a need to understand how DD has been used as a methodological tool for the co-design, modifications, implementation, evaluation, and knowledge dissemination of health promotion interventions. This scoping study aims to comprehensively understand the application of DD in intervention design to provide a framework to ensure DD is employed with methodological rigour. It will offer valuable insights into how its systematic use can improve the credibility, validity, and trustworthiness of study findings while respecting the principles of participation and knowledge co-production.

**Methods:**

This scoping review follows the Arksey & O’Malley framework. The Arksey & O’Malley framework is designed to map the key concepts, types of evidence, and gaps in research, consisting of five stages: identifying research questions, selecting relevant studies, screening, data charting, and summarizing results. The research team includes decision-makers, researchers, healthcare providers involved in the co-design, co-implementation and co-evaluation of health-promoting interventions, and two patient partners with previous experience in collaborative decision-making. Searches will be performed across multiple databases such as OVID Medline, PsycINFO, PubMed, CINAHL, and Scopus databases. Studies will undergo abstract and full-text screening using Covidence. Covidence is an online platform designed to simplify the process of creating systematic and other in-depth literature reviews (including scoping reviews, rapid reviews, and meta-syntheses), abstract, full-text screening, and extraction of study details, results, and references. A data extraction template has been co-developed building on Guidance for Reporting Involvement of Patient and Public (GRIPP2), which ensures comprehensive reporting of patient and public involvement in research, and the Consolidated Standards of Reporting Trials (CONSORT) checklist facilitates the consistent reporting of methodologies. This data will allow us to understand how DD is used to co-design health interventions. Data extraction will be performed by one reviewer and verified by a second reviewer for consistency. It will then be synthesized to map how DD has been used across various stages of health promotion interventions.

**Ethics and dissemination:**

This scoping review does not require ethics approval as it analyzes data from existing research articles. The results will inform the development of guidelines to support methodologically rigorous DD regarding the co-design, co-implementation, and co-evaluation of health-promoting interventions.

**Supplementary Information:**

The online version contains supplementary material available at 10.1186/s40900-025-00680-9.

## Abstract (plain language summary)

Deliberative dialogue (DD) is a structured research approach that brings together diverse voices—such as patients, healthcare professionals, and researchers—to discuss complex health challenges and collaboratively develop solutions. As the need for inclusive and participatory health research grows, DD is increasingly used to co-design, implement, and evaluate health promotion initiatives. However, there is limited understanding of how DD is applied across different stages of health interventions.To address this gap, a team of researchers, healthcare providers, and patient partners is conducting a scoping review to analyze how DD has been used in health promotion research. This review will map out its applications, identify strengths and challenges, and offer insights into how it can be used effectively to improve health outcomes. By examining existing studies across multiple academic databases, the team aims to provide recommendations for using DD in a methodologically rigorous way.The findings of this study will contribute to the development of guidelines to support researchers and decision-makers in employing DD for equitable and impactful health interventions. The results will be shared through academic publications, presentations, and accessible materials co-created with patient partners to ensure broad dissemination.

**Supplementary Information:**

The online version contains supplementary material available at 10.1186/s40900-025-00680-9.

## Introduction

Deliberative dialogue (DD) is a participatory research methodology that brings stakeholders from diverse backgrounds together to share opinions and exchange solutions to complex issues [1]. DDs involve integrating both research evidence and the lived experiences of diverse stakeholders to facilitate knowledge exchange, foster mutual understanding, broaden perspectives, and build consensus where possible [1]. With participatory methods being heavily encouraged, including by funding agencies such as the Canadian Institutes of Health Research CIHR [2] and National Institutes of Health (NIH) [3] and journals such as the Canadian Medical Association Journal CMAJ [4] and British Medical Journal (BMJ) Open [5], it is becoming increasingly important to distinguish and clarify the terms used to describe multistakeholder approaches in health research. While co-production and Patient and Public Involvement (PPI) are action-oriented processes—with co-production emphasizing equitable partnerships and PPI involving advisory roles for patients and the public with varying degrees of decision-making power—DD is a methodology designed to support these processes through its structured focus on dialogue, argumentation, and reflection.

The increasing use of participatory methods in health research [6–15] highlights the need for clarity, as DD offers a distinct framework that complements and enhances collaborative efforts. Unlike general collaborative processes, DD provides a structured and adaptable methodology that can be tailored to the specific research needs, enabling researchers to engage stakeholders effectively in addressing complex, value-laden issues. Examples of DD approaches (see Table 1) include Citizens’ Juries, which empower the public to deliberate on health policy decisions, and *Choosing HealthPlans All Together (CHAT)*, whichhelps stakeholders prioritize healthcare benefits. DD has also been applied in priority-setting across various levels of healthcare, from macro-level policy discussions to micro-level service improvements [16–18].

DD has been commonly used in health policy research and is increasingly used within health promotion. According to the WHO’s Ottawa Charter, created in 1986, health promotion is defined as “enabling people to increase control over and to improve their health” [19]. The charter lays down three prerequisites, namely ‘advocate,’ ‘enable,’ and ‘mediate’ as foundations for health promotion [19, 20]. DD embodies these principles, enabling individuals to take control over and ‘advocate’ for their health while fostering collective and coordinated action to ‘mediate’ diverse priorities and build consensus.

Several rhetorical dimensions characterize DD, essential for facilitating well-structured, reasoned discussions. As outlined by Friess and Eilders (2015), these dimensions frame how DD participants engage in deliberation:

1. Rationality: Participants are expected to present well-reasoned arguments, providing logical justifications for their claims. This dimension ensures that discussions remain evidence-based, leading to more productive and relevant discourse [21].
2. Interactivity: Refers to the engagement between participants, including structured elements like turn-taking and more cognitive aspects, such as attention to the contributions of others. This ensures that dialogue is dynamic, with participants actively responding to one another [22].
3. Equality: Ensures that all participants have an equal opportunity to voice their opinions and respond. This inclusivity is critical in health promotion, where diverse stakeholders, including marginalized voices, must be heard.
4. Civility: Refers to maintaining a respectful and polite tone throughout the dialogue. Civility ensures that discussions remain constructive, facilitating cooperation rather than conflict [23].
5. Common Good Reference: Participants are encouraged to frame their arguments regarding the collective benefit, ensuring that the focus remains on outcomes that serve the broader community rather than individual interests.
6. Constructiveness: Dialogue is expected to be solution-oriented, with participants approaching the conversation sincerely and with the intention of resolving issues. This constructiveness is particularly relevant in addressing complex health challenges.

In health promotion, DD provides an effective framework for tackling “wicked problems” [11, 24]—complex, multifaceted health issues that lack clear solutions [25]. By facilitating collaborative problem-solving, DD helps define and tackle these problems in a participatory and inclusive manner [26]. An example of this can be seen in the work of the Mental Health Commission of Canada, which employed DD techniques to develop the country’s first mental health strategy, addressing national mental health priorities in a collaborative, stakeholder-driven process [11, 24]. Despite the gradual increased use of DD, there remains a gap in understanding how DD is applied explicitly as a tool for co-design, co-implementation, and co-evaluation of health-promoting interventions. Existing literature has not comprehensively explored the structured ways in which DD can advance equity and inclusivity—core tenets of health promotion—across different stages of intervention development. This scoping review aims to address a gap in the lack of comprehensive mapping of how Deliberative Dialogue (DD) has been applied specifically in the co-design, co-implementation, and co-evaluation of health-promoting interventions by exploring how DD has been used in the co-design, co-implementation, and co-evaluation stages of health interventions. By synthesizing existing evidence, this review will provide insights into the methodologies, outcomes, and challenges associated with DD, while identifying areas for future research. It will also provide actionable insights for researchers, healthcare providers, and decision-makers seeking to employ participatory methods. The findings will contribute to advancing the understanding of participatory approaches in health promotion and their potential to improve collaborative decision-making. To our knowledge, this will be the first review to systematically explore DD’s role in developing health promotion interventions, with results expected to inform the creation of a standardized DD methodology for future research. Given that DD is still evolving globally, we anticipate the number of relevant studies may be limited in some regions. If most of the identified literature originates from Canada, the US, or the UK, we will treat this as a limitation and discuss how it may affect how it can be applied to the broader context. Despite the possible limitations, examining these studies will help highlight common DD processes, identify barriers, and propose ways to strengthen DD practice.

**Table 1:** Deliberative dialogue (DD) approaches

DD Approaches	Description
1. Citizen Juries	A Citizens’ Jury is a deliberative process involving a small, randomly selected group of citizens representing a specific area’s demographics. These jurors come together to discuss and deliberate on a particular policy issue, aiming to reach a collective decision or recommendation [27].
2. Priority Setting	Also known as rationing or resource allocation, it is the process of determining how to distribute limited resources within health systems at various levels—macro (e.g., government), meso (e.g., regional health authorities and hospitals), and micro (e.g., clinical programs)—through deliberation [28].
3. Deliberative Democracy	A system where people discuss issues together before making decisions. For it to be democratic, everyone should have equal decision-making power, usually represented by one vote per person, although smaller groups might use other methods. To be considered deliberative, there must be equity in participation, lack of coercion and censorship, and engagement with disagreement [27].
4. Deliberative Transnationalism	An approach that emphasizes collaborative decision-making in governance. It combines principles of deliberative democracy with transnational governance, focusing on how public participation and expert deliberation can enhance the legitimacy and effectiveness of decision-making processes across countries [29].

## Methods

### Protocol design

We will adhere to a research approach aligned with the Preferred Reporting Items for Systematic Reviews and Meta-Analysis extension for Scoping Reviews. Methodologically, scoping reviews are instrumental in assessing the breadth and depth of knowledge within a specific field. They facilitate the development of new insights by searching for, selecting, and synthesizing key concepts, ideologies, and gaps in the literature without focusing on the quality of the published works. Documenting this protocol is an important step in outlining our scoping review plan. This paper will utilize the methodological framework proposed by Arksey and O’Malley (2005) framework, incorporating the recommendations proposed by Levac et al. (2010) [29, 30] detailed in the study methodology below. The PRISMA-Scr checklist (see Supplementary Material 1) will also enhance the quality of the scoping review [30]. The PRISMA-Scr Checklist is a tool that provides guidelines for reporting key elements of a scoping review and systematic review to ensure transparency and completeness [31].

The scoping study aims to Identify and map existing studies that use Deliberative Dialogue (DD) in health-promoting interventions, describe how DD is employed at various stages of co-production (design, adaptation, implementation, evaluation, and dissemination), and evaluate the extent to which DD approaches incorporate equity, address power imbalances, and include marginalized voices [32].

#### Role of patient partners in the study

Patient partners have been involved since the inception of this study, contributing to the design, development of research questions, and overall methodological approach to align the study with practical realities and lived experiences. Their input has strengthened the relevance and inclusivity of decisions about which studies to include, including shaping the inclusion and exclusion criteria. During data extraction and analysis, patient partners will play a key role in interpreting the findings, helping to ensure that the identified themes and conclusions resonate with those directly impacted. In the dissemination phase, patient partners will co-create materials with the research team; these will include infographics, summaries, and presentations tailored to diverse audiences, including communities, practitioners, and policymakers. Their involvement will with the research team accessible, actionable, and relevant to those who may benefit from the findings of this scoping study.

### Study methodology

#### Stage 1: identifying the research question

The scoping review aims to answer the following questions:

1. How has DD been applied at each intervention stage (co-design, co-implementation, and co-evaluation)?
2. What are the unique contributions of DD at each stage, including process, outcomes, and challenges specific to those phases?
3. What is the role of diverse stakeholders, including those with lived/living experience, at different stages of intervention development?
4. What was the rationale for using DD in studies of health interventions?

These questions were developed collaboratively with the involvement of patient partners and the research team. During the initial planning stages, discussions were held to establish the research questions and the objectives of the scoping study.

#### Stage 2: identifying relevant studies

The search strategy for the scoping review was developed after consultation with a librarian from the University of Toronto. The finalized search strategy was peer-reviewed, and suggestions were provided to improve its comprehensiveness. The finalized strategy implemented in Medline can be seen in Supplementary Material 2 and was adapted to be utilized in various other databases. This scoping review included studies from OVID Medline, OVID PsycInfo, Pubmed, EBSCO CINAHL, and Scopus, with the last search conducted in June 2024. Primary and secondary peer-reviewed articles were included in the review.

#### Stage 3: selecting studies

The search results will be exported to Covidence for deduplication, referencing, screening, and data extraction. Covidence is an online platform designed to simplify the process of creating systematic and other in-depth literature reviews (such as scoping reviews, rapid reviews, and meta-syntheses) [33]. Covidence facilitates tasks like citation screening, full-text screening and review, extracting study details and results, and exporting data and references. A two-stage screening process will begin with abstract and title screening, followed by full-text screening. The abstract and title screening will be performed by two reviewers who can choose *‘yes*,*’ ‘no*,*’ or ‘maybe’* to indicate whether they meet the inclusion criteria. Screening conflicts will be resolved by a third reviewer in consultation with the two reviewers. Before initiating the screening, the reviewers screened a small number of randomly sampled articles from the search results to ensure the minimum rate of inter-rater reliability was met [34]. While the abstract screening phase would include systematic and scoping reviews, the full-text screening phase will only include the primary research papers included in these reviews that meet the inclusion criteria. Importantly, papers using DD methods to develop policy interventions were excluded as they have been extensively studied in the literature [7, 35, 36]. The inclusion and exclusion criteria for the review can be seen in Table 2.

**Table 2:** Scoping review inclusion and exclusion criteria

Criteria	Inclusion	Exclusion
Participants	• Individuals impacted by the problem (patients, caregivers, family members) • Individuals with the power to address the problem (researchers, clinicians, policymakers, leaders, decision-makers)	
Interventions	• Health Promotion - Optimizing opportunities for health and wellbeing (physical and psychosocial) through health information, preventive programs, and access to medical care.	Policy Interventions
Concepts	Deliberative dialogue is a group process that can help integrate and interpret scientific and contextual data. It may also be called deliberative democracy, dialogue processes, talking circles, sharing circles, etc.	
Types of sources	Empirical literature	Non-Empirical & Grey Literature
Design/date	Papers published after the year 2000	
Language	English	Literature available only in other languages

#### Stage 4: extracting data

Data from the selected publications will be extracted and organized based on the data extraction Template (Table 3) for the Intervention Description and Replication (GRIPP2) checklist and the Consolidated Standards of Reporting Trials (CONSORT). Initially, data will be descriptively organized from the literature, including information on the target population, intervention characteristics, and effectiveness measures reported. The study team will then identify and extract the intended and unintended, as well as the positive and negative impacts of each intervention. Team members will independently extract the first three articles and then come together as a larger group to address any discrepancies. The remaining articles will be assigned for data extraction, and the full team will convene for regularly scheduled reviews to facilitate the process. Any outstanding discrepancies will be resolved collaboratively by team members.

**Table 3:** GRIPP2 and CONSORT Checklist

	**Domain Name**	**Description**
**GRIPP 2**	Publication title	Title of publication
	Author’s name	Name of study authors. If there are more than three authors, list the first author’s last name followed by ‘et al.’
	Year of publication	The year the study was published.
	Study location	The location geographical location where the study was conducted (i.e., Canada, UK, USA, etc.).
	Proposed aim of the study	Describe any rationale, theory, or goal of the elements essential to the intervention.
	Theoretical underpinning	Describe the theoretical rationale and any theoretical influences relating to PPI/Stakeholder involvement/and DD in the study.
	Design	A clear description of how the stakeholders/PPI were involved in the DD (PWLEs, policymakers, etc.) and how equity was considered. Outlines the number of dialogues held and the rationale for using DD.
	People involved	A clear description of patients, caregivers, the public, or community members involved with the PPI activity in the study and the number of people involved in the DD.
	Stages of involvement	Report on how PPI/Stakeholders were Involved and how DD was used at different stages of the study.
	Level of involvement	Reports on the level or nature of PPI/Stakeholder Involvement/DD at various stages of the study.
	Qualitative evidence of impact	Report on the methods used to qualitatively explore the impact of PPI/Stakeholder/DD in the study (if applicable)
	Quantitative evidence of impact	Reports on the methods used to quantitatively measure or assess the impact of PPI/Stakeholder involvement in the DD (if applicable)
	Robustness of measure	Reports on the rigour of the method used to capture or measure the impact of PPI/Stakeholder Involvement in the DD (if applicable)
	**Domain**	**Description**
**CONSORT**	Outcome	Defines prespecified primary and secondary outcome measures, including how and when they were defined, monitored and assessed.
	Effectiveness	Identifies whether the intervention was successful in increasing uptake of the intervention or other related outcome measures.
	Limitation	Identifies limitations of the intervention, including potential bias and imprecision. Reported strengths and limitations of the DD methodology used.
	Generalisability	External validity and applicability of the study findings.
	Interpretation	Interpretations consistent with results, balancing benefits and harms, and considering other relevant evidence

#### Stage 5: collating summarizing and reporting the outcomes

The synthesis will involve descriptive summaries from each domain (see Table 3) and thematic analysis to collate the findings. The data will include publication details such as titles, authors, and study locations, and describe interventions’ aims, theoretical underpinnings – if any, and target populations. Stakeholder involvement will be explored, focusing on how patients, public participants, and other stakeholders contributed to DD processes across study stages (see Table 3). Attention will be paid to methodological rigour using tools such as GRIPP2 and CONSORT checklists to evaluate the quality and robustness of the included studies. Descriptive themes will highlight methodologies, limitations, and the outcomes of DD processes, while analytic themes will focus on understanding DD’s rationale, effectiveness, and potential limitations. Transparency and consistency in reporting will be guided by the PRISMA-ScR checklist (See Supplementary Material 1).

The GRIPP2 short-form checklist was utilized to report patient and public or community partners’ involvement through DD in research [37]. The GRIPP2 checklist, increasingly adopted for reporting PPI in primary and secondary research, will ensure our analysis comprehensively addresses research question 2 [37]. To ensure the quality of data reporting, the PRISMA-ScR checklist will be employed. See Supplementary Material 1.

Study findings will be disseminated through a peer-reviewed journal, posters, and infographics shared with patient partners and stakeholder communities. The manuscript and other final products will be co-created with the stakeholders involved in our study.

## Dissemination

Study findings will be disseminated through a peer-reviewed journal, posters, and infographics shared with patient partners and stakeholder communities. The manuscript and other final products will be co-created with the patient partners and other stakeholders involved in our study.

The review’s findings will be prepared into a guideline document to support methodological best practices for other researchers seeking to use DD as a tool for co-production. We will disseminate it broadly in an interdisciplinary way with the support of our project partners, including people with lived experience.

## Electronic supplementary material

Below is the link to the electronic supplementary material.

Supplementary Material 1

Supplementary Material 2

## Data Availability

No datasets were generated or analysed during the current study.
