# Correction to: Deliberative dialogue for co-design, co-implementation and co-evaluation of health-promoting interventions: a scoping review protocol

**DOI:** 10.1186/s40900-025-00698-z

**Published:** 2025-03-14

**Authors:** Kian Godhwani, Abimbola K. Saka, Vinesha Ramasamy, Bee-Lee Soh, Mathangee Lingam, Aisha Lofters, Eva Grunfeld, David Gerstle, Peter Selby, Ambreen Sayani

**Affiliations:** 1 Women’s College Research Institute, Women’s College Hospital, Toronto, ON Canada; 2 Campbell Family Mental Health Research Institute, Centre for Addictions and Mental Health, Toronto, ON Canada; 3 Department of Family and Community Medicine, University of Toronto, Toronto, ON Canada; 4 Dalla Lana School of Public Health, University of Toronto, Toronto, ON Canada; 5 University of Toronto Mississauga, Library, Mississauga, ON Canada

**Correction to: Res Involv Engagem 11**,** 16 (2025)**

10.1186/s40900-025-00680-9

Following publication of the original article [1], the authors identified an error in the author name of Eva Grunfeld and the patient partners weren’t indicated.

The incorrect author name is: Eva Grundfield.

The correct author name is: Eva Grunfeld.

Vinesha Ramasamy and Bee-Lee Soh are patient partners.

The author group has been updated above and the original article [1] has been corrected.
