# Supplementary figures and images for: Deliberative dialogue for co-design, co-implementation and co-evaluation of health-promoting interventions: a scoping review protocol
Source: Res Involv Engagem. 2025 Feb 28;11:16. doi: 10.1186/s40900-025-00680-9 (PMC11869413; doi:10.1186/s40900-025-00680-9)

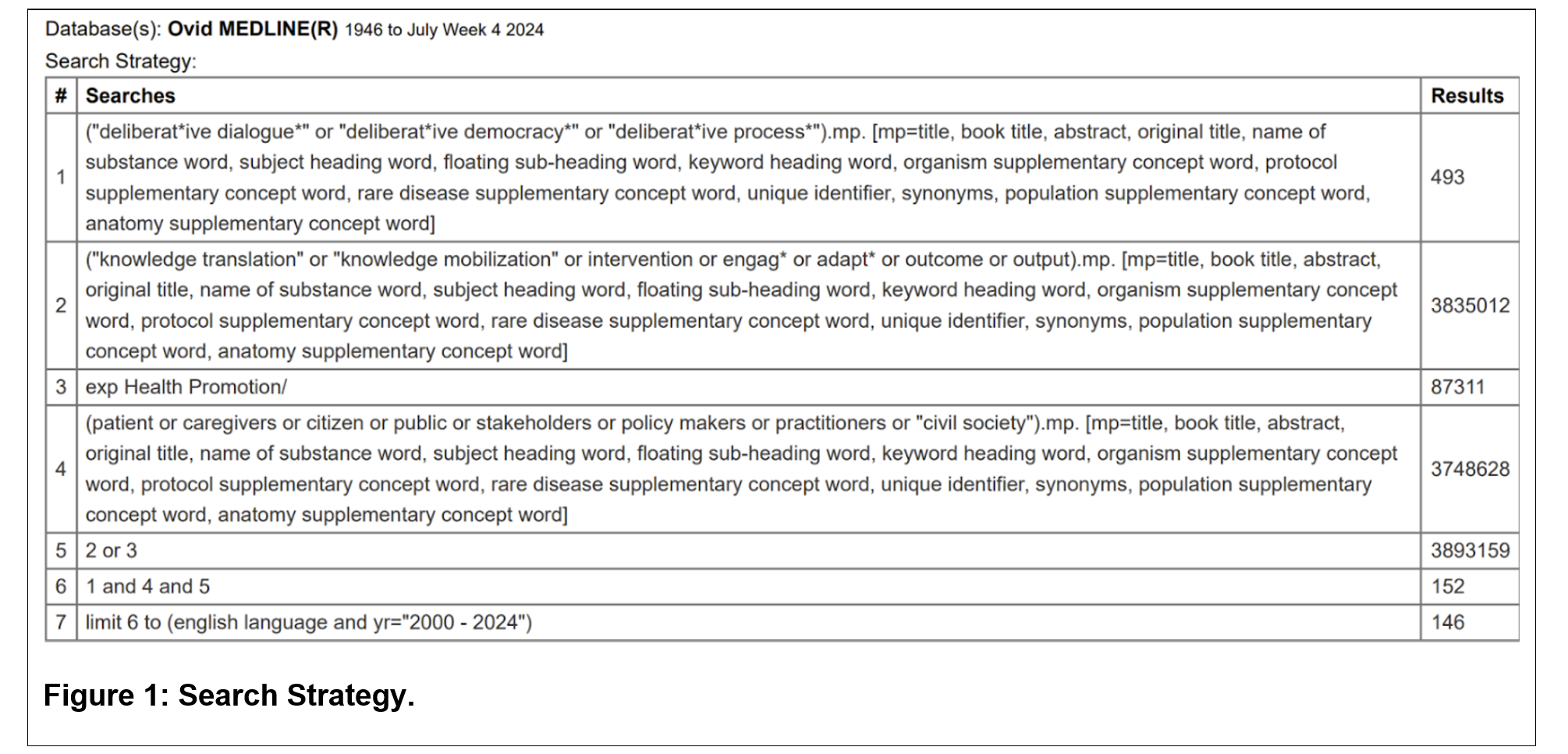

Supplement: Supplementary file 2 — Supplementary Material 2 [file 40900_2025_680_MOESM2_ESM.png]
